# Directional migration of mesenchymal stem cells under an SDF-1α gradient on a microfluidic device

**DOI:** 10.1371/journal.pone.0184595

**Published:** 2017-09-08

**Authors:** Siwan Park, Hwanseok Jang, Byung Soo Kim, Changmo Hwang, Gi Seok Jeong, Yongdoo Park

**Affiliations:** 1 Department of Biomedical Engineering, Biomedical Science of Brain Korea 21, College of Medicine, Korea University, Seoul, Korea; 2 Department of Biomedical Science, Graduate School of Medicine, Korea University, Seoul Korea; 3 Biomedical Engineering Research Center, Asan Institute for Life Sciences, Asan Medical Center, Seoul, Korea; The University of Akron, UNITED STATES

## Abstract

Homing of peripheral stem cells is regulated by one of the most representative homing factors, stromal cell-derived factor 1 alpha (SDF-1α), which specifically binds to the plasma membrane receptor CXCR4 of mesenchymal stem cells (MSCs) in order to initiate the signaling pathways that lead to directional migration and homing of stem cells. This complex homing process and directional migration of stem cells have been mimicked on a microfluidic device that is capable of generating a chemokine gradient within the collagen matrix and embedding endothelial cell (EC) monolayers to mimic blood vessels. On the microfluidic device, stem cells showed directional migration toward the higher concentration of SDF-1α, whereas treatment with the CXCR4 antagonist AMD3100 caused loss of directionality of stem cells. Furthermore, inhibition of stem cell’s main migratory signaling pathways, Rho-ROCK and Rac pathways, caused blockage of actomyosin and lamellipodia formation, decreasing the migration distance but maintaining directionality. Stem cell homing regulated by SDF-1α caused directional migration of stem cells, while the migratory ability was affected by the activation of migration-related signaling pathways.

## Introduction

Stem cell homing is a controlled recruitment of stem cells within the vascular endothelium that leads to trans-endothelial and directional migration. Damaged tissues in heart, liver, and other organs can be regenerated by stem cell homing through well-directed migration of stem cells. The directional migration of stem cell is precisely regulated by the homing factors released from the injury sites. The released soluble cytokines, homing factors, contribute to generating the cytokine gradient that determines the direction of stem cell migration. Consequently, the bio-chemical gradient induces stem cells to migrate to the injury site for regeneration.

**Fig 1:**
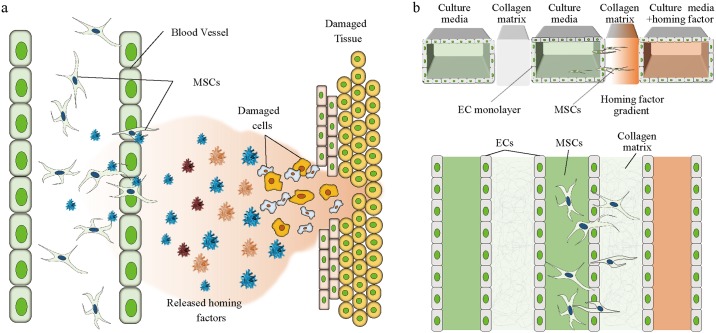
Mesenchymal stem cell homing mimicked on a microfluidic device. (a) Theoretical schematic of peripheral MSC homing process. (b) Illustration of the microfluidic device and stem cell homing on-chip.

Although the healing process by stem cells has not been elucidated, it has been shown that homing factors have a pivotal role in tissue regeneration [1]. After tissue damage, homing factors such as SDF-1α also known as the C-X-C motif chemokine 12 (CXCL12) is released from the damaged site. A predominant receptor for the SDF-1α is CXCR4 which is a seven transmembrane G protein-coupled receptor widely expressed in cells and tissues taking a role in vasculogenesis and organogenesis [2, 3]. More importantly, down regulation of CXCR4 and SDF-1α significantly decreased the invasiveness of cancer cells, meaning that expression of CXCR4 is responsible for the cell recruitment [4, 5]. CXCR7 is also a protein known as the receptor of SDF-1α [2, 6]. The released homing factors form a chemical gradient from the injury site to the surrounding area, which initiates the transmigration of stem cells through the endothelium and directional migration into the stromal tissue (Fig 1a) [7]. Dar *et al* have shown enhanced trans-endothelial migration under a gradient of SDF-1α [8]. Cheng *et al* showed that stem cells overexpressing CXCR4, contributes to the improvement of cardiac performance in myocardial infarction [9], illustrating that SDF-1α is a key homing factor for stem cells [10]. However, the mechanism behind the directional migration of mesenchymal stem cells (MSC) through the endothelium due to a chemokine gradient has not been clearly elucidated in *in vivo* or conventional *in vitro* experimental systems.

Directional migration of stem cells during stem cell homing is a key mechanism of homing from the blood vessels to injury sites based on the gradient of homing factors. Peripheral MSCs expressing CXCR4 are trafficked by the gradient of SDF-1α. Binding of SDF-1α leads to activation of signaling pathways related to migratory mechanisms such as Rho-ROCK, Rac, and Cdc42 [11]. Rho-ROCK and Rac pathways are known for their roles in the synthesis of migratory machineries for the cells and are mediated by SDF-1α ligand binding [12, 13].

Although there are limitations in the study of microfluidics [14], the device used in this study is a fascinating system that is able to mimic numerous *in vivo* microenvironments generating gradients of soluble cytokines. Directional migration was incorporated into a collagen matrix-integrated microfluidic device, which could be used for the assessment of stem cell homing [15] (Fig 1b). Chung *et al* developed the basic three-channel-based microfluidic chips with collagen matrix as a barrier of fluid on a vascular structure [16]. Also, the capability of this device to form endothelial cell (EC) monolayers allowed the observation of EC migration and sprouting through the collagen matrix by Chung *et al* and Jeong *et al* [16, 17].

To better understand the homing mechanism, Boyden chambers and transwells have been used as tools to observe the increased migration of MSCs due to the chemokine effect of SDF-1α [1, 18]. However, none of these devices showed the chemotaxis of MSCs through the endothelial barrier or the ECM conditions, meaning that the devices were not able to mimic the *in vivo* spatial environment. The proposed device has a geometric set up for building perfusable vessel structures as well as the ECM environment and has both biological and technical advantages over the Boyden chamber and the transwell systems. Furthermore, the previous studies did not focus on the effect of migratory inhibitors during cellular migration or on the quantification of migration distance or directional migration.

In this study, we describe the directional migration of MSCs under a gradient of homing factors using a microfluidic channel. To identify the behaviors of homing factors, directional movement and transmigration of MSCs were observed. To construct an in-vivo-mimicking microenvironment, an EC barrier was constructed by forming an EC monolayer along the center channel of the microfluidic device. Directionality and migratory ability of MSCs were assessed in the presence of different inhibitors. Five conditions including the control environment were created by exposing inhibitors to MSCs undergoing migration; (i) Control group without SDF-1α gradient, (ii) SDF-1α condition, (iii) AMD3100 (CXCR4 antagonist) treatment condition, (iv) Y-27632 (Rho-ROCK inhibitor) treatment condition, and (v) NSC23766 (Rac inhibitor) treatment condition. The inhibitor AMD3100 was used for blocking SDF-1α binding to CXCR4 in order to disrupt the directionality of MSC and Y-27632, and NSC23766 were used to disable the migratory mechanism of the stem cells. Treatment with Y-27632, an inhibitor of the Rho-ROCK signaling pathway, and NSC23766, an inhibitor of the Rac signaling pathway, resulted in decreased migration distance of MSCs without loss of directionality. In contrast, the CXCR4 antagonist AMD3100 disrupted the directionality of the MSCs but did not affect the migration ability of the stem cells resulting in near average migration distances.

## Material and methods

### 2.1 Chip fabrication

#### 2.1.1. Microfluidic device

**Fig 2:**
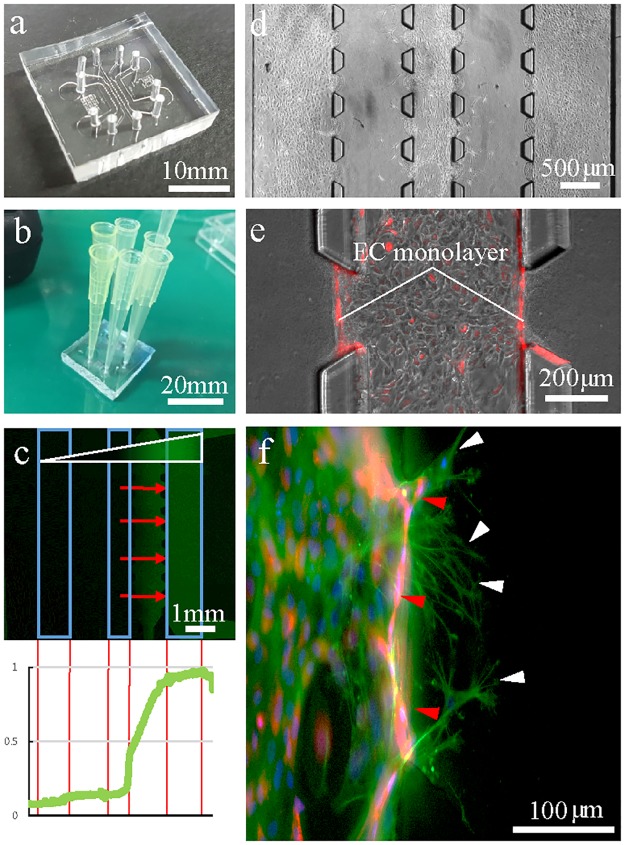
Extravasation of MSCs under a gradient of homing factor. **(a) An image of the PDMS-based microfluidic device**. (b) The microfluidic device set up for cell seeding. Micropipette tips were used as a media reservoir. (c) A FITC-dextran gradient within the collagen matrix on a microfluidic device with endothelial monolayers (blue squares) embedded in cell channels. Red arrows indicate the direction of MSC migration due to an SDF-1α gradient. The graph indicates the fluorescence intensity of dextran within the chip. (d) A microscopic image of MSC extravasation and migration through the collagen matrix on a microfluidic device. (e) RFP-tagged endothelial cells are used to distinguish migrating MSCs. (f) MSC extravasation. The white arrow indicates MSCs, and red arrow indicates endothelial cells.

To visualize the directional migration of MSCs, a polydimethylsiloxane (PDMS; Sylgard 184A, B Dow Chemical, MI, USA) device (Fig 2a) inspired by a microfluidic chip used in a previous study was fabricated [16]. Using conventional soft lithography, PDMS (Sylgard A: Sylgard B = 10:1) was cured on an SU-8 (MicroChem, MA, USA) wafer and placed at 80°C. An individual chip had a 25mm width and length and a post-molding height of 250μm. The center cell seeding channel was 0.5mm wide, and the two side cell seeding channels were 1mm wide. Each of the collagen gel channels was 1mm wide (S1b Fig). After curing the microfluidic channel, the device was autoclaved and bonded with cover slips under oxygen plasma treatment. Within 10 minutes after bonding, 1mg/ml Poly-D-Lysine (PDL; Sigma-Aldrich, St. Louis, MO) was applied to the inside of the chip for enhancement of adhesion of collagen as well as the EC monolayer. After at least two hours of incubation with PDL coating, the channels were washed with triple-distilled water and dried in an oven for 24 hours.

#### 2.1.2 Cell seeding and settings of the microfluidic device

The main set up for this experiment is inhibition of EC migration during induced stem cell migration. Metabolic balance is controlled by consumption of nutrients and oxygen in the culture media in each channel. Equal numbers of ECs were seeded in all three channels for equal consumption of nutrients and oxygen in culture media, allowing the endothelial cells to remain as a stable monolayer. On the other hand, MSCs are set to migrate toward a higher concentration of metabolic factors from the side channels. In this experiment, we compare the numbers of MSCs migrating both toward and away from the SDF-1α gradient in order to identify the cells migrating under the influence of SDF-1α.

### 2.2 Evaluation of gradients of the microfluidic chip

#### 2.2.1. Collagen gel preparation

In order to prepare the collagen gel, stock type I collagen (BD Bioscience, MA), 10X PBS (Gibco BRL, NY, USA), filtered triple-deionized water, and 0.5N NaOH were kept in an ice bath and mixed to a final pH of 7.4 and concentration of 2.5mg/ml. Just after mixing, collagen solution was carefully introduced into the ECM channels of the microfluidic chip. Then, the chip was placed in the humidified chamber and placed in the incubator at 37°C for 30 min. Next, 37°C warmed media was applied in order to prevent dehydration or shrinkage of the collagen matrix.

#### 2.2.2. SDF-1α gradient

Two hours after MSC seeding, cell culture medium containing 250ng/ml of SDF-1α (R&D Systems, MN, USA) was applied only to the conditioning channel to generate an SDF-1α gradient within the collagen gel matrix. The concentration of SDF-1α *in vivo* is known to be about 0.5 to 0.8 ng/ml in human circulation and 1 to 5 μg/ml in *in vivo* fluids. However, the optimal concentration range for most chemokines for *in vivo* cell attraction is 10 to 1000 ng/ml [19]. Here, 250ng/ml of SDF-1α was applied every 24 hours after washing the channels with media in order to reset the gradient.

#### 2.2.3. Dextran gradient test

To ensure that the chemokine gradient is sustained in the collagen matrix, 10 kDa fluorescence isothiocyanate (FITC)-dextran (FITC-dextran, 10kDa, Sigma-Aldrich, St. Louis, MO) was applied to visualize the gradient when an endothelial monolayer was present (Fig 2c). 10 kDa FITC-dextran was used because of its identical molecular weight to SDF-1α. Fluorescence images were taken every 4 hours for up to 44 hours. The dextran gradient was renewed 24 hours after the initial creation. Image J (NIH image, Wayne Rasband) was used for plot profiling and measuring the fluorescence intensity of dextran. The maximum fluorescent intensity (1.0) was shown to slightly decrease over time; plot profiling clearly illustrated the level of the gradient. However, the intensity also seemed to fluctuate while decreasing because the least intense fluorescence is not at the final hours. However, the intensity generally decreased by about 20 to 50% within the collagen matrix throughout the experiment. This evidence suggests that the SDF-1α gradient is sustained within the collagen gel for chemokine-derived migration of MSCs (S3 Fig). The consumption rate of SDF-1α is not well studied; therefore, the results might vary due to the reduction of chemokines in certain areas and deformation of the gradient. However, this graphic data is strong support that the gradient is not saturated within the collagen and sustains the homing process for over 40 hours.

### 2.3 Directional migration of stem cells under physiological conditions

#### 2.3.1. Cell culture

Red fluorescent protein (RFP) expressing human umbilical vein endothelial cells (RFP-HUVECs) (Olaf Pharmaceuticals, Inc., USA) were cultured in endothelial cell growth medium (EGM TM-2) (Lonza, MD, USA) supplemented with 5% FBS, 0.04% hydrocortisone, 0.4% hFGF-B, 0.1% VEGF, 0.1% R3IFG-1, 0.1% ascorbic acid, 0.1% hEFG, and 0.1% GA-1000. The seeding concentration of RFP-HUVECs in the microfluidic channel was 2 x 10^6^ cells/ml. Human originated mesenchymal stem cells (hMSCs) (Lonza, MD, USA) were cultured in Dulbecco’s modified Eagle’s media (DMEM; Gibco BRL, NY, USA) supplemented with 10% FBS and 1% (v/v) antibiotics. A cell suspension with an MSC concentration of 4.5 x 10^5^ cells/ml was used for microchip injection. MSCs were not used after their ninth passage. Both cells were cultured in regular 75T cell culture plates before they were used in the chip.

RFP-HUVECs were seeded twice at a 2-hour interval in order to seed the cells thoroughly in the channels. The collagen gel interface was endothelialized by seeding HUVECs in the main channels. After seeding, the chips were tilted at various angles and placed for 30 minutes to form a uniform EC monolayer (S5 Fig). After 2 to 3 days of culturing EC in the microfluidic device, monolayer formation could be seen under the microscope (Fig 2d and 2e), and MSCs were introduced in the middle channel with different conditioned media. Media was composed of EGM-2 and DMEM in a ratio of 2:1 and was changed every 24 hours. For cell culture, micropipette tips were used as a media reservoir (Fig 2b).

#### 2.3.2. Treatment with cell migration inhibitors

In order to influence the migration of MSCs, AMD3100 (CXCR4 antagonist), Y-27632 (inhibitor of Rho-ROCK pathway), and NSC23766 (inhibitor of Rac pathway) were applied throughout all channels. Each of the inhibitors Y-27632 (25μM, Sigma-Aldrich, St. Louis, MO, USA), NSC23766 (50μM, Sigma Aldrich, St. Louis, MO, USA), and AMD3100 (25μg/ml, Sigma Aldrich, St. Louis, MO, USA) were mixed in 2:1 EGM-2 and DMEM with addition of SDF-1α (250 ng/ml) on the condition channel. A total of five groups including conditions with media only (no SDF-1α, no drugs), SDF-1α only group, and three inhibitor-treated groups were tested. Media for each group were replaced every 24 hours, and images were obtained with an EVOS fluorescence microscope (EVOS^®^ FL Auto, Life Technologies, USA).

### 2.4 Assessment of stem cell homing in microfluidic chips

#### 2.4.1. Stem cell migration analysis

Images were obtained with EVOS (EVOS^®^ FL Auto) every 24 hours for 2 days. Migrating cells were counted with Image J software, and after immunohistochemistry, the distance of migration was measured from the starting point of the collagen matrix to the nucleus of the cell with Image J. Confocal images were taken with an LSM710 (Zeiss, Germany) and analyzed with ZEN Black Edition.

Stem cells migrated from the endothelial monolayer were counted on EVOS microscopic images. Cells were distinguishable since the cytosol of ECs was expressing RFP and MSCs were not. MSCs in the process of transmigration were also counted for extravasated cells since their branches were within the collagen matrix (Fig 2f).

Cell migration displacement was measured by DAPI images obtained also with an EVOS microscope. The distance measurement was performed utilizing both the RFP and DAPI images to distinguish between MSCs and RFP-ECs. The term displacement is used because cells do not migrate in straight lines, although the measurement was in the linear distance. The distance was measured from the start of the collagen matrix or endothelial monolayer to the nucleus of the stem cells with Image J.

#### 2.4.2. Immunohistochemistry

The channels of the microfluidic chip were gently washed with PBS to remove all media. Then, 4% paraformaldehyde was applied and cooled to 4°C. When the cells were fixed, F-actin was labeled with Alexa Fluor 488 phalloidin (Thermo Fisher Scientific Cat# A12379, RRID:AB_2315147), and nuclei were stained with 4’6-diamidino-2-phenylindole (Thermo Fisher Scientific Cat# D1306, RRID:AB_2629482). Cells were permeabilized with 0.1% Triton X-100 (Sigma-Aldrich, St. Louis, MO, USA) for 30 minutes. After permeabilization, BSA treatment was applied for 30 minutes, after which phalloidin and DAPI dissolved in BSA were applied to all channels. After two hours of staining, the channels were washed with 1X phosphate buffered saline tween-20 (PBST) to remove the fluorescent noise.

VE-cadherin was stained with Anti-VE Cadherin antibody—Intercellular Junction Marker (Abcam Cat# ab33168, RRID:AB_870662) and Alexa Fluor 594 goat anti-rabbit lgG (H+L) (Molecular Probes Cat# A-11012, RRID:AB_141359) (Life Technologies, USA) after being fixed with 4% paraformaldehyde.

## Result and discussion

### 3.1. Chip fabrication and gradient formation

Homing factors are released from an injury site and form a chemokine gradient across the surrounding area to induce MSC migration toward the injury site (Fig 1a). When SDF-1α is bound to CXCR4, the associated MSCs migrate toward the higher end of the gradient. To mimic this event in a microenvironment, the microfluidic device was re-designed and modified from a previously reported version [16]. The differences in device design are 5 times more regions of interest (ROIs) and wider gel channel areas for observation of the migration tendency (Fig 1b). The microfluidic device used in this experiment has three main cell seeding channels separated by two gel channels. (S1a Fig). The three main channels are designed to provide both control and experimental conditions in one chip sample. Also, the separating collagen scaffolds help divide the three main channels to ensure that they are physically independent. Since the device allows cell culture inside the channels, we co-cultured ECs with MSCs for observation of transendothelial migration (Fig 1b).

Stem cell migration due to the chemokine effect of SDF-1α has been previously demonstrated on a Boyden chamber. Imitola *et al* showed increased stem cell migration through a fibronectin-coated membrane depending on the dosage of SDF-1α [18]. The Boyden chamber is a useful tool to examine chemotaxis [20] but has limitations in spatiotemporally mimicking the *in vivo* environment. The proposed microfluidic device, however, has significance in mimicking the spatial environment with both a chemokine gradient to initiate directional migration and inhibitors to affect the migratory mechanisms of stem cells. Applications of different environmental conditions on the chip produced significantly different results, which provided insights into cellular migration depending on the chemical environment.

The main set up for this experiment was to inhibit the migration of ECs while inducing stem cell migration. Differential migration was achieved by controlling the consumption of nutrients in the culture media in each channel [17]. Equal numbers of ECs were seeded in all three channels, which led to equal consumption of the culture media and maintenance of the endothelial cells as a stable monolayer (S2c and S2d Fig). If only one of the channels was seeded, ECs would start sprouting toward the higher concentration of metabolic factors by degrading the collagen gel matrix (S2a and S2b Fig). Conversely, MSCs were seeded only in the center channel in order to easily initiate the migration toward the higher concentration of metabolic factors from the side channels. In this experiment, we compared the numbers of MSCs migrating both toward and away from the SDF-1α knowing that the migration is both caused by the metabolic and chemokine gradient. However, when both sides were compared, more of MSCs were estimated to migrate towards the chemokine gradient.

In order to simulate the gradient formation of SDF-1α (10kDa) in microfluidic channels, FITC-dextran of same molecular weight was used for generating a visual gradient. Maximum fluorescent intensity (1.0) was slightly declining over time and with plot profiling, the fluorescent intensity data was analyzed. The intensity was generally decreasing throughout the experiment by about 20 to 50% within the collagen matrix. This evidence suggested that the SDF-1α gradient was sustained within the collagen gel for chemokine derived migrations of MSCs (S3 Fig).

**Fig 3:**
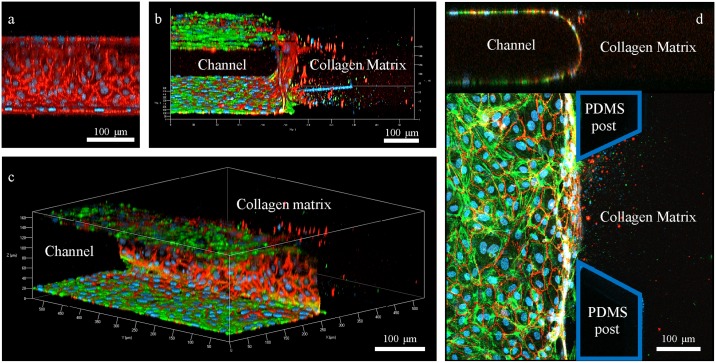
Confocal microscopic images of endothelial monolayer formed within the channel of the microfluidic device. (a) Side view of the endothelial monolayer confluent with collagen matrix. RFP represents the expression of VE-cadherin and blue represents DAPI. (b) Front view of the endothelial monolayer confluent with both the channel and the collagen matrix. GFP represents expression of actin fibers within the cells. (c) Overall view of the endothelial monolayer confluent within the channel of the microfluidic device. (d) Ortho view of the endothelial monolayer. The top image shows homogeneous confluence of cellular monolayer formed throughout the channel. Blue territory represents the PDMS posts.

A uniform confluence of the endothelial monolayer at the interface with the collagen matrix and the channel surface was a crucial factor in preventing undesirable leakage of the chemokine gradient in the microfluidic devices. VE-cadherin (vascular endothelial-cadherin) is a glycoprotein responsible for cell-cell adhesion, whose expression contributes greatly to endothelial permeability by regulating intercellular junctions [21]. Significant expression of VE-cadherin means that the cells are well-adhered to form a strong barrier. VE-cadherin expressed in seeded endothelial cells in the microfluidic device reflects the formation of a definite EC monolayer which was observed under the confocal microscopy (Fig 3a–3c). The expression level of VE-cadherin confirms that the endothelial monolayer constructed within the microfluidic device is reliable (Fig 3d).

To ensure that the endothelial monolayer in the microfluidic device had barrier functions, the plot profile was used to measure the intensity of the FITC-dextran fluorescence over 16 hours with and without the EC monolayer. The result showed stabilized fluorescence intensity in the presence of an EC monolayer. On the other hand, the dextran gradient seemed to randomly flow within the device, showing a significant increase in fluorescence intensity in the ROIs (n = 4, p<0.05) (S4b and S4c Fig). This indicates that the EC monolayer within the device performed a barrier-like role, acting like a dam on a river to stabilize the gradient of the chemokine.

### 3.2. Extravasation and directional migration of stem cells under a chemokine gradient

Fig 2f shows the extravasation of MSCs in the microfluidic channel integrated with an EC monolayer. During the homing process, MSCs stimulated by SDF-1α within blood vessels coordinately bind to the endothelium for the initiation of transendothelial migration. MSCs roll to bind to endothelial cells via P-selectin and VCAM-1/VLA-4, expressed by endothelial cells when the homing factors are released under shear flow [22]. In this device, however, the shear flow is absent in order to observe the more efficient directional migration of MSCs. Furthermore, the shear flow would disrupt the uniform gradient formation of SDF-1α within the collagen matrix as well as in the channels. It is unclear, in this device, if the MSCs were bound via a P-selectin- and VCAM-1/VLA-4-dependent method for extravasation. A future study should focus on developing the conditions for MSC adhesion on the endothelial monolayer and on the spontaneous formation of an SDF-1α gradient in the presence of shear flow.

**Fig 4:**
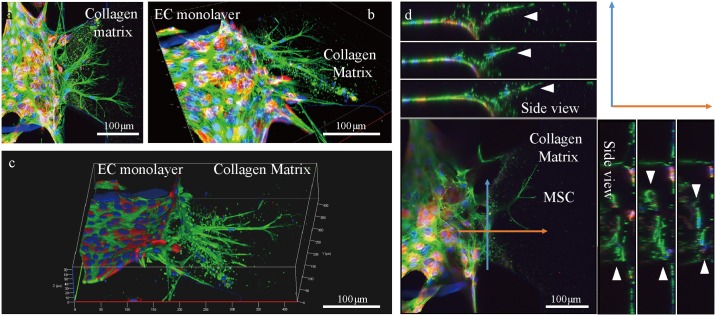
3D confocal images of MSCs migrating transendothelially. (a-c) Red fluorescence shows the cytosol of RFP-tagged EC. Green fluorescence shows actin fibers of both MSCs and ECs. Cells without red fluorescence are MSCs. Blue are nuclei of the cells stained with DAPI. (d) Ortho view of transendothelial migration of MSCs. Blue and orange arrows indicate the side view range of the directions of movement. White arrows indicate the locations of nuclei of migrating MSCs.

Transendothelial migration of MSCs during homing occurs in three dimensions *in vivo*. In order to ensure that MSCs were migrating into the 3D collagen matrix in the microfluidic device, confocal images were taken (Fig 4a–4c). MSCs and ECs could be distinguished by the ECs expressing RFP in their cytosol. The ortho images showed that the nucleus of the MSC is not on the surface of the chip, but in the middle of the collagen gel (Fig 4d). This implies that the cells are migrating without the help of a rigid surface to grab onto but are solely migrating within the collagen matrix, as MSCs would migrate through the ECM in an *in vivo* environment. This is possibly due to the PDL coating, which enhanced the adhesion of collagen matrix while triggering 3-dimensional migration of cells [23].

Stem cell homing is initiated by the gradient of SDF-1α secreted from injury sites. SDF-1α binds to its cellular receptor CXCR4, which triggers signaling pathways resulting in a variety of events such as chemotaxis and proliferation via gene transcription [24]. CXCR4-mediated chemotaxis is mediated by PI3 kinase (PI3K), which is activated by the Gβγ and Gα subunits that were separated when the ligand was bound [25].

**Fig 5:**
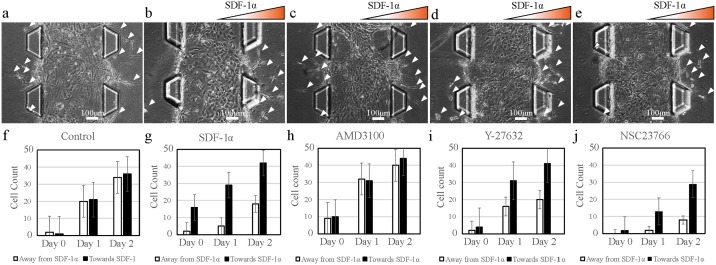
Numbers of extravasating and directionally migrating MSCs in the direction of the SDF-1α gradient under different conditions. (a) Control group; no inhibitor and no SDF-1α gradient. *p>0.05 (b) MSCs under SDF-1α gradient *p<0.05 (c) MSCs under AMD3100 (CXCR4 antagonist) treatment. *p>0.05 (d) MSCs under Y-27632 (Rho-ROCK inhibitor) treatment *p<0.05. (e) MSCs under NSC23766 (RAC inhibitor) treatment *p<0.05. (f-j) Graph of numbers of extravasated MSCs moving toward and away from the SDF-1α gradient. Y-axis represents numbers of cells, and X-axis represents number of days. White arrows indicate the extravasated MSCs. Paired t-test was applied for number of MSCs migrating on each side for each conditions with n = 8.

According to the test, about 66% of total extravasated MSCs migrated through the collagen matrix toward the higher concentration of SDF-1α (Fig 5g) (n = 8, p<0.05). Extravasations and migrations away from the gradient were observed due to possible metabolic factor gradient also initiating the migration (Fig 5b). However, more migration toward the gradient clearly indicates that SDF-1α mediates stem cell homing and migration. This result was supported by the present results of the control group where no SDF-1α was applied to the MSCs. Almost equal numbers of MSCs extravasated and migrated without an intended direction (Fig 5a and 5f) (n = 5 p<0.3). This result indicates that most of the stem cells seeded in the central channel are moving toward a higher SDF-1α gradient. The direction of cell migration through chemokine in this microfluidic experiment may be direct evidence of homing of MSCs *in vivo*.

**Fig 6:**
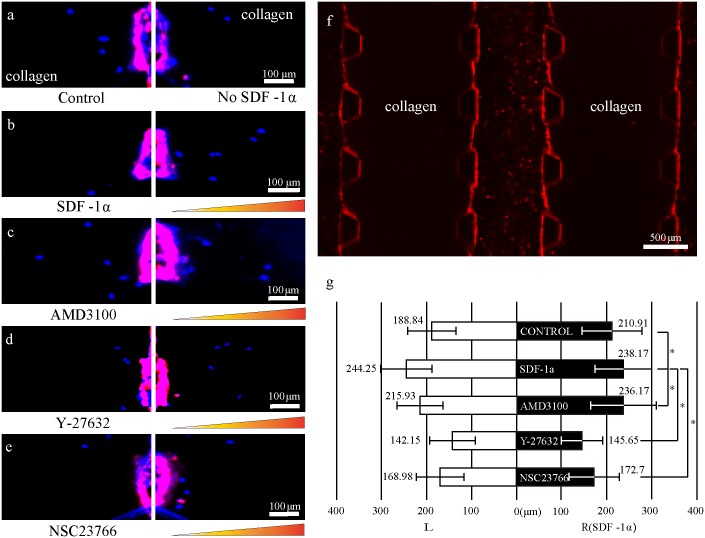
Migration distance of MSCs over the duration of the experiment. **Blue dots in the collagen matrix indicate the locations of nuclei of MSCs** (a) Control (b) SDF-1α condition. *p>0.05 (c) AMD3100-treated condition. *p>0.05 (d) Y-27632-treated condition. *p<0.05 (e) NSC23766-treated condition. *p<0.05 (f) RFP-expressing endothelial monolayer confluent to the collagen matrix. RFP-expressing ECs are distinguishable from MSCs. (g) Average migration distance of individual MSCs toward or away from the SDF-1α gradient. Graph bars from top to bottom; Control, SDF-1α, AMD3100, Y-27632, NSC23766. Purple and red fluorescence represents endothelial cells. The monolayer near the collagen matrix is shown in purple because of the higher population of ECs along the Z-axis. Orange triangle represents the presence of SDF-1α gradient. F-test was used to compare the average migration of each conditions with the control group. *p<0.05 represents significant variance difference while *p>0.05 represents no significant variance difference. One-way ANOVA analysis for finding inequality group showed *p<0.05.

Treatment with the CXCR4 antagonist AMD3100 on the stem cell homing chip resulted in a lack of directionality in cell migration (n = 8, p<0.03). AMD3100 is known to block the binding of monoclonal antibodies to CXCR4, preventing SDF-1α-CXCR4 binding [26–28]. Theoretically, inhibition of the SDF-1α-CXCR4 interaction would result in MSC failure to detect the gradient. With no exceptions, migration data (Fig 5c and 5h) showed identical results to the control group. This result suggests that SDF-1α is a crucial factor for directional migration of MSCs. MSCs treated with AMD3100 did not show any decrease in migration distance compared to SDF-1α and control group, indicating that AMD3100 does not influence the cellular migratory mechanisms (Fig 6c) (n = 8, p<0.05). AMD3100 is a well-known factor for diminishing the directional cellular migration caused by the chemical guidance [29], but there is no significant evidence that AMD3100 influences the migration distance of MSCs.

### 3.3. Effects of Rho-ROCK inhibitors and Rac inhibitors on directional migration during stem cell homing

One of the most important parts of stem cell homing is the mechanism by which the cells migrate. Migration of stem cells is a coordinated process where multiple signaling pathways activate physical machineries of the cells to migrate [30, 31]. The migration distance of MSCs after extravasation from the endothelial monolayer was measured to determine the possible conditions that disrupt the MSC homing process. The migration distance also provides an insight into the motility mechanism contributing to the migration of MSCs or any type of migrating cells. The most representative signaling pathways related to cell migration are Rho-ROCK and Rac signaling pathways, and this study provides conditions influencing these mechanisms [32, 33].

The Rho-ROCK signaling pathway is responsible for cytoskeleton regulation such as stress fiber assembly, actomyosin contraction, and actin membrane linkage [34, 35]. The reagent known to block this pathway is Y-27632, which reduces a cell’s ability to migrate by constraining the regulation of the cytoskeleton [36]. Treatment with Y-27632 (25 μM) did not block the binding of SDF-1α to CXCR4, resulting in normal directional migration toward the chemokine gradient (Fig 5d and 5i) (n = 8, p<0.05). However, treatment with Y-27632 did produce a significant decrease in the migratory ability of the MSCs since their migration displacement was the smallest of all conditions (~145 μm) (Fig 6d) (n = 8, p<0.05). This migration displacement is about 40% less than that of the SDF-1α group (Fig 6a) and 28% less than the control group (Fig 6b). Adamson *et al* showed in a previous study that treatment with Y-27632 to endothelial cells did not cause a notable disruption in endothelial barrier properties even though the formation of actin fibers was blocked [37]. Therefore, the decreased distance of migration was not affected by the endothelial barrier properties.

The Rac signaling pathway is responsible for lamellipodia protrusion of the cell [38, 39]. Lamellipodia are like grappling hooks used to grab the ECM scaffold and drag it in the direction of migration. Once the hook is attached, contraction of acto-myosin by the Rho-ROCK pathway is activated for migration in the intended direction. Blocking this signaling pathway prevents cells from attaching to the collagen matrix and partially restricts the migratory mechanisms. Treatment with NSC23766, an inhibitor of the Rac signaling pathway, showed a slight decrease in migration distance (Fig 6e) (n = 5, p<0.05). Again, the SDF-1α gradient was still effective to cause directional migration (Fig 5e and 5j) (n = 5, p<0.05). This result points out that although migratory potential had been lowered due to inhibiting Rac signaling pathway [40] the directional migrations of stem cells were still preserved by the chemokine effect.

Spindler *et al* showed that cAMP plays a role in stabilizing the endothelial barrier via GTPase Rac1 activation. According to the study, disrupting Rac1 activation caused the endothelial barrier to lose its stability and increase its permeability [41]. Theoretically, the number of extravasating MSCs in our experiment should have been increased due to lack of endothelial barrier integrity after treatment with NSC23766. However, the results actually showed a decreased number of extravasated MSCs regardless of endothelial barrier integrity, possibly because of the simultaneous influence of NSC23766 on the MSCs.

### 3.4. Migration distance vs. morphology of MSCs

**Fig 7:**
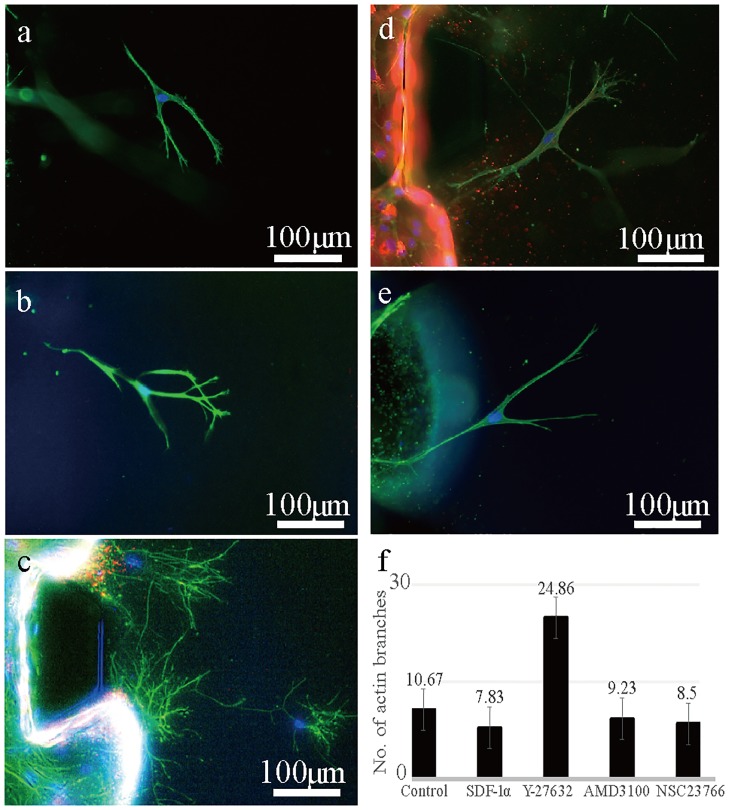
Morphology of F-actin-stained MSCs under different conditions (branch counting). (a) Morphology of MSCs without SDF-1α gradient. (b) Morphology of MSCs under the gradient of SDF-1α. (c) Morphology of MSCs under Y-27632 treatment (d) Morphology of MSCs under AMD3100 treatment (e) Morphology of MSCs under NSC23766 treatment (f) An average number of actin branches of MSCs counted with Image J. One-way ANOVA analysis was used for branch counting. *p<0.05 represents at least one group has inequality.

Migration distances were measured to test the migratory ability of stem cells when exposed to different chemical conditions. The migration distance was able to be measured because ECs were distinguishable from MSCs due to their expression of RFP in the cytosol (Fig 6f). The average migration distances were produced after measuring the displacement of all the individual migrating MSCs in the collagen matrix. The migration distance of MSCs under Y-27632-treated conditions was the shortest compared to SDF-1α conditions (Fig 6g). A potential reason for this could be the decreased actin organization governed by Rho-ROCK signaling pathways. As expected, F-actin staining of Y-27632-treated MSCs had shown unusual morphologies. Blocking the Rho-ROCK pathway seemed to disrupt the formation of strong and firm stress fibers, negatively affecting forward cellular movement. MSCs expressed thin and hair-like protrusions (24.86 branches per cell) (Fig 7c) instead of forming the thick and strong actin fibers (~10 branches per cell) that were observed in the other conditions (Fig 7a, 7b, 7d and 7e). Furthermore, actin fibers seemed to be dispersed without any directional pattern, which indicates that cells were not readily migrating towards the direction as they intended. The result indicated that well-coordinated actin fiber formation is one of the key governors for the cellular migration. Also, bundles of actin fibers are bound together in the direction of chemokine signaling to generate the powerful motility force.

The morphology of NSC23766-treated stem cells showed a normal distribution of actin fibers (Fig 7e), like all other conditions (Fig 7a–7c) except the Y-27632-treated (Fig 7c) condition. However, the migration distance of MSCs under NSC23766 was slightly decreased (n = 5, p<0.05). This is due to lack of lamellipodia and filopodia formation by the Rac pathway. Despite this, the distance decrease in the NSC23766 condition is not as significant as that of cells under the Y-27632 condition, which indicates that actin fiber coordination is more important than lamellipodia formation for cellular migration.

**Fig 8:**
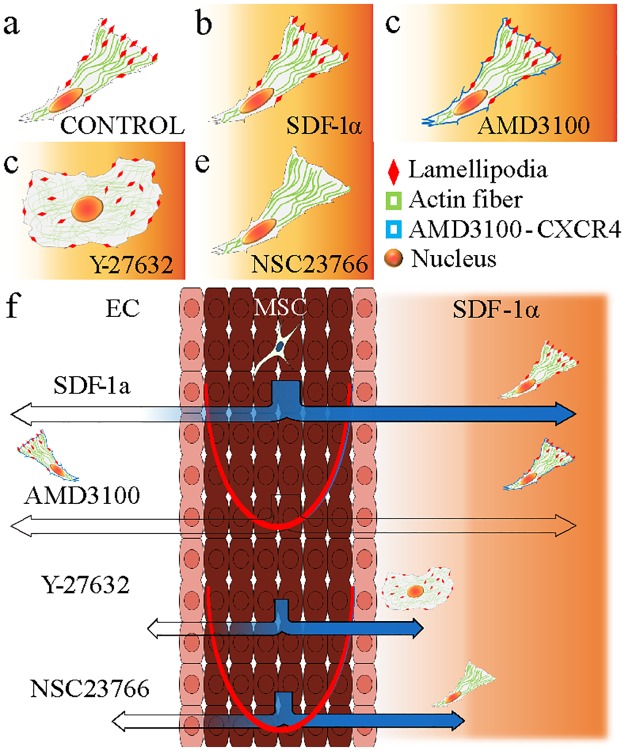
Illustration of extravasation and directional migration of MSCs in different conditions. (a) Morphology of a normal MSC. (b) An MSC under SDF-1α gradient. (c) An MSC exposed to Y-27632, a Rho-ROCK inhibitor. (d) An MSC exposed to AMD3100, a CXCR4 antagonist. (e) MSC exposed to NSC23766, a Rac inhibitor. (f) Migration of MSCs due to the chemokine effect of SDF-1α. Blue color represents the number of stem cells migrating in response to the homing factor.

These experiments with the proposed microfluidic device quantitatively demonstrated the possible mechanisms of directional migration and homing of stem cells *in vivo* and are in agreement with previous studies (Fig 8) [42–44]. MSCs exposed to five different conditions showed different migratory behaviors, related to the expression level of migratory machineries such as actin fibers, filopodia, and lamellipodia (Fig 8a–8e). With a lack of actin fiber formation, the MSCs are not able to exert the strong forces needed to produce fast move. Also, failure to form lamellipodia would also slow the cellular migration. However, the directionality of migration in both cases would be preserved as long as SDF-1α successfully binds to CXCR4 of the stem cells (Fig 8f).

Cell migration is a complex biochemical process including polarization, adhesion and protrusion, and rear retraction. In order for the series of events to occur, numerous signaling pathways are activated to coordinate the cell migration. In this study, two of the major determinants of cell migration (Rho-ROCK and Rac) had been chosen to create conditions that could influence the driving force of MSCs. Disordering the actin fiber formation via inhibiting the Rho-ROCK signaling pathway was the most effective way to slow down the migration of MSCs. Furthermore, this microfluidic platform still has a potential for more sophisticated control of microenvironment and chemical conditions for further studies.

## Conclusion

Stem cell homing is a crucial biological event that plays important roles in wound healing and tissue regeneration. This experiment showed the implementation of stem cell homing on a PDMS-based microfluidic device. This microfluidic device could provide different conditions such as a chemical gradient affecting the behavior of cells. The exact difference in cellular behavior between homing and normal migration is unknown. However, the level of MSC migration towards the SDF-1α gradient was greater than the number of MSCs away from it and it is clear that SDF-1α plays an important role in MSC homing. MSCs on a microfluidic device migrated for various reasons, such as chemical and metabolic gradients, but showed a more sensitive reaction to the chemokine effect. CXCR4 antagonist disrupted the detection of chemotaxis for MSCs and led them to migrate without directionality. Rho-ROCK inhibitor plays a critical role in inhibiting the formation of strong cellular fibers, resulting in decreased migration distance; a similar effect was shown with a Rac inhibitor that inhibited the formation of lamellipodia and filopodia. The inhibitors used in this experiment showed observable effects on stem cell migration; however, in order to generate more dramatic influences, further study of combined application of inhibitors is required. Although stem cell homing was successfully implemented *in vitro*, there are still some challenges for setting the perfect conditions to mimic *in vivo* conditions because there are additional factors that influence the migration of stem cells such as EC monolayer junction integrity, flow conditions, and combined use of inhibitors.

## Supporting information

S1 Fig Dimensions of the microfluidic device used in this study. (a) Specifications of the PDMS-based microfluidic device. (b) Three main cell seeding channels divided by collagen channels. Notice that the collagen channel is open throughout its length, providing more regions of interests. (Post height: 250μm).
(TIF)

S2 Fig Angiogenic sprouting of ECs due to the metabolic gradient formed by the three channels. (a) 4X view of EC sprouting; only the center channel is seeded with ECs, creating a chemical gradient due to consumption of nutrients. Radical sprouting of ECs within the collagen matrix. (b) 10X view of EC sprouting; the EC monolayer fails to adhere to the collagen matrix (c) Balanced metabolic gradient after every channel is seeded with ECs; no sprouting takes place (d) EC monolayer is stabilized and maintained. Extravasation and directional migration of MSCs in different morphologies and conditions.
(TIF)

S3 Fig FITC-dextran gradient test on a microfluidic device with an EC monolayer and a collagen matrix embedded. (a) First 24 hours of FITC-dextran gradient test within the collagen matrix on a microfluidic device with endothelial cells embedded. The fluorescent intensity was measured every 4 hours. (b) After the first 24 hours, the channels were washed with media and refilled with dextran at the same concentration. The intensity was measured for another 24 hours.
(TIF)

S4 Fig FITC-dextran gradient intensity level at the region of the endothelial monolayer confluent on the collagen gel and collagen gel without an endothelial monolayer embedded. (a) The microfluidic device with a FITC-dextran gradient. White dashed box shows the region of interest for plot profiling of fluorescence intensity. Areas A and B are chosen to test the effects of endothelial barrier properties. (b) Gradient intensity at point A is shown on a graph. Notice that the fluorescent intensity is more stable when an endothelial monolayer is embedded. (c) Gradient intensity at Point B is shown on a graph, demonstrating a similar trend of gradient formation to Point A.
(TIF)

S5 Fig Endothelial cell seeding and formation of endothelial monolayers on a microfluidic device. (a) ECs were introduced in the microfluidic device which was initially positioned upright. (b) The microfluidic device was skewed for endothelial monolayer’s confluence with collagen matrix. (c) The microfluidic device was skewed in the opposite direction for endothelial monolayer’s confluence with collagen matrix on both sides. The microfluidic device was kept in each position for 30 minutes each.
(TIF)
