# Supplementary figures and images for: Directional migration of mesenchymal stem cells under an SDF-1α gradient on a microfluidic device
Source: PLoS One. 2017 Sep 8;12(9):e0184595. doi: 10.1371/journal.pone.0184595 (PMC5590985; doi:10.1371/journal.pone.0184595)

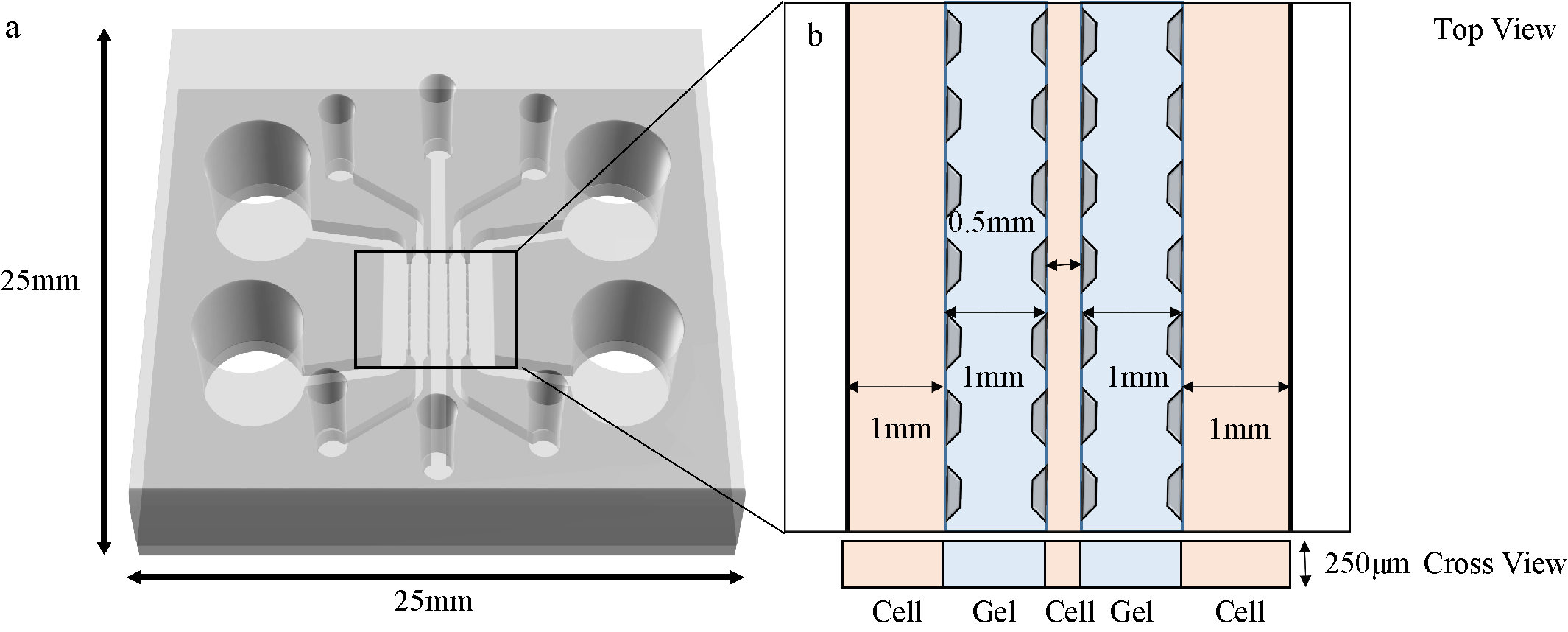

Supplement: S1 Fig — (a) Specifications of the PDMS-based microfluidic device. (b) Three main cell seeding channels divided by collagen channels. Notice that the collagen channel is open throughout its length, providing more regions of interests. (Post height: 250μm). (TIF) [file pone.0184595.s001.tif]

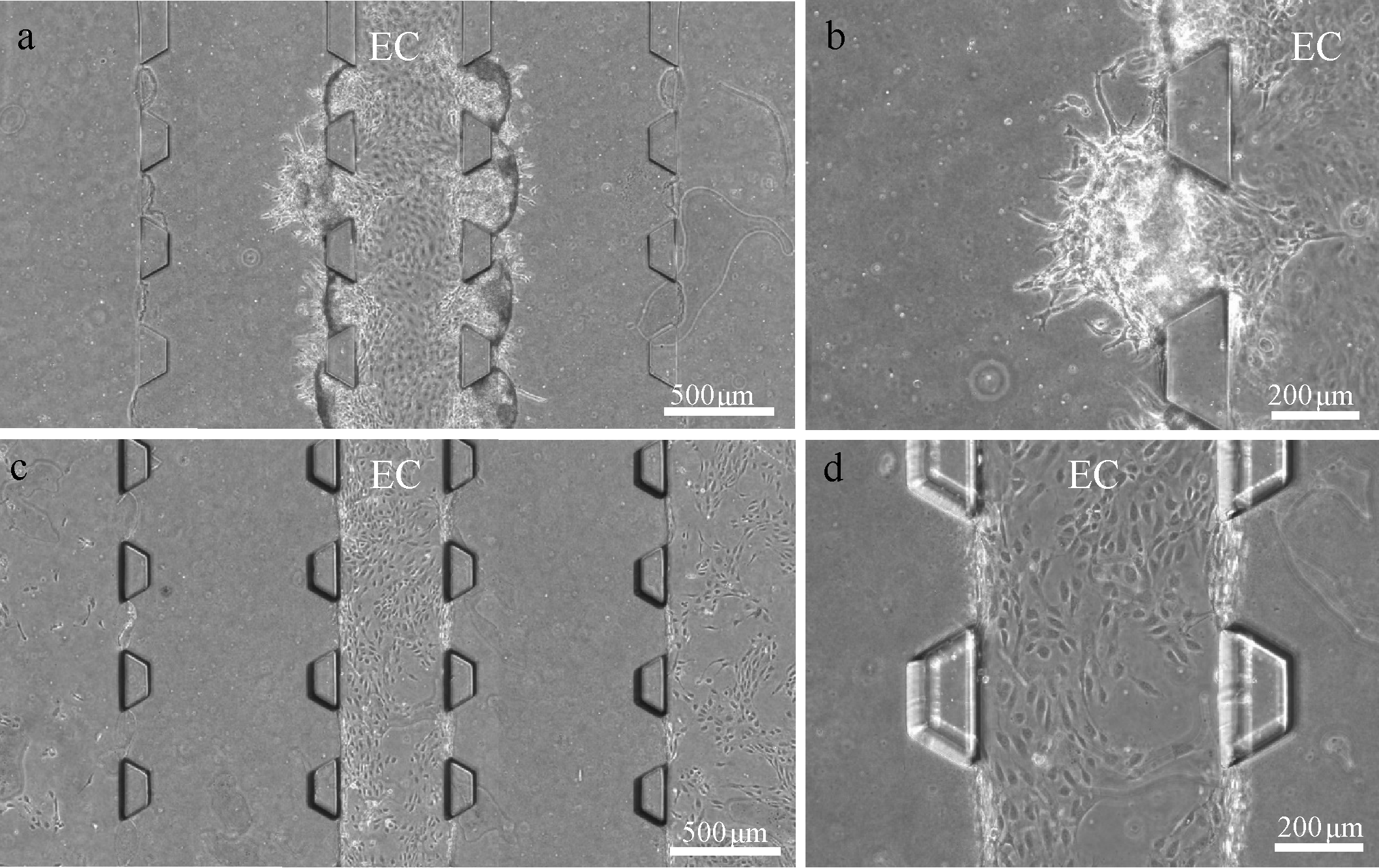

Supplement: S2 Fig — (a) 4X view of EC sprouting; only the center channel is seeded with ECs, creating a chemical gradient due to consumption of nutrients. Radical sprouting of ECs within the collagen matrix. (b) 10X view of EC sprouting; the EC monolayer fails to adhere to the collagen matrix (c) Balanced metabolic gradient after every channel is seeded with ECs; no sprouting takes place (d) EC monolayer is stabilized and maintained. Extravasation and directional migration of MSCs in different morphologies and conditions. (TIF) [file pone.0184595.s002.tif]

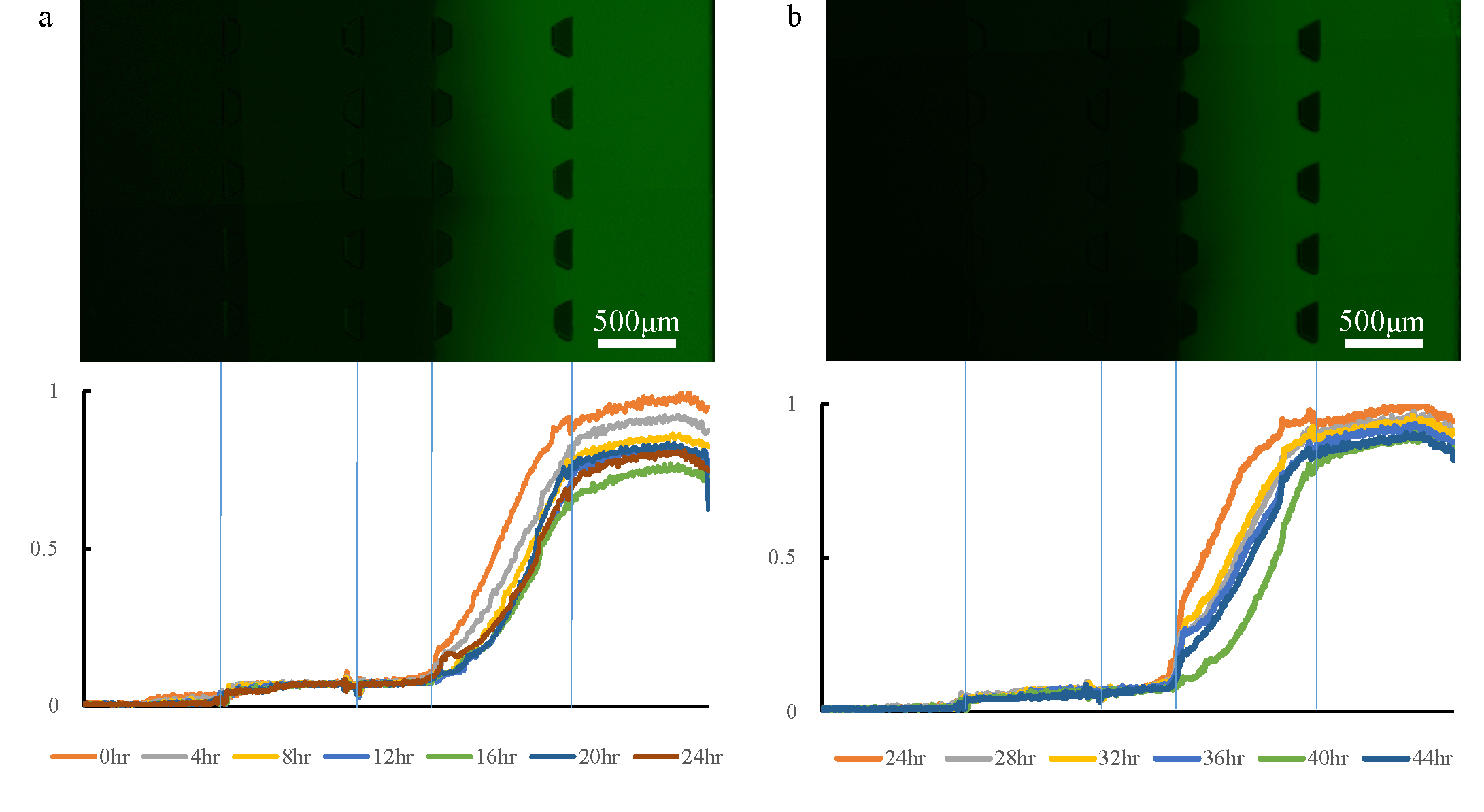

Supplement: S3 Fig — (a) First 24 hours of FITC-dextran gradient test within the collagen matrix on a microfluidic device with endothelial cells embedded. The fluorescent intensity was measured every 4 hours. (b) After the first 24 hours, the channels were washed with media and refilled with dextran at the same concentration. The intensity was measured for another 24 hours. (TIF) [file pone.0184595.s003.tif]

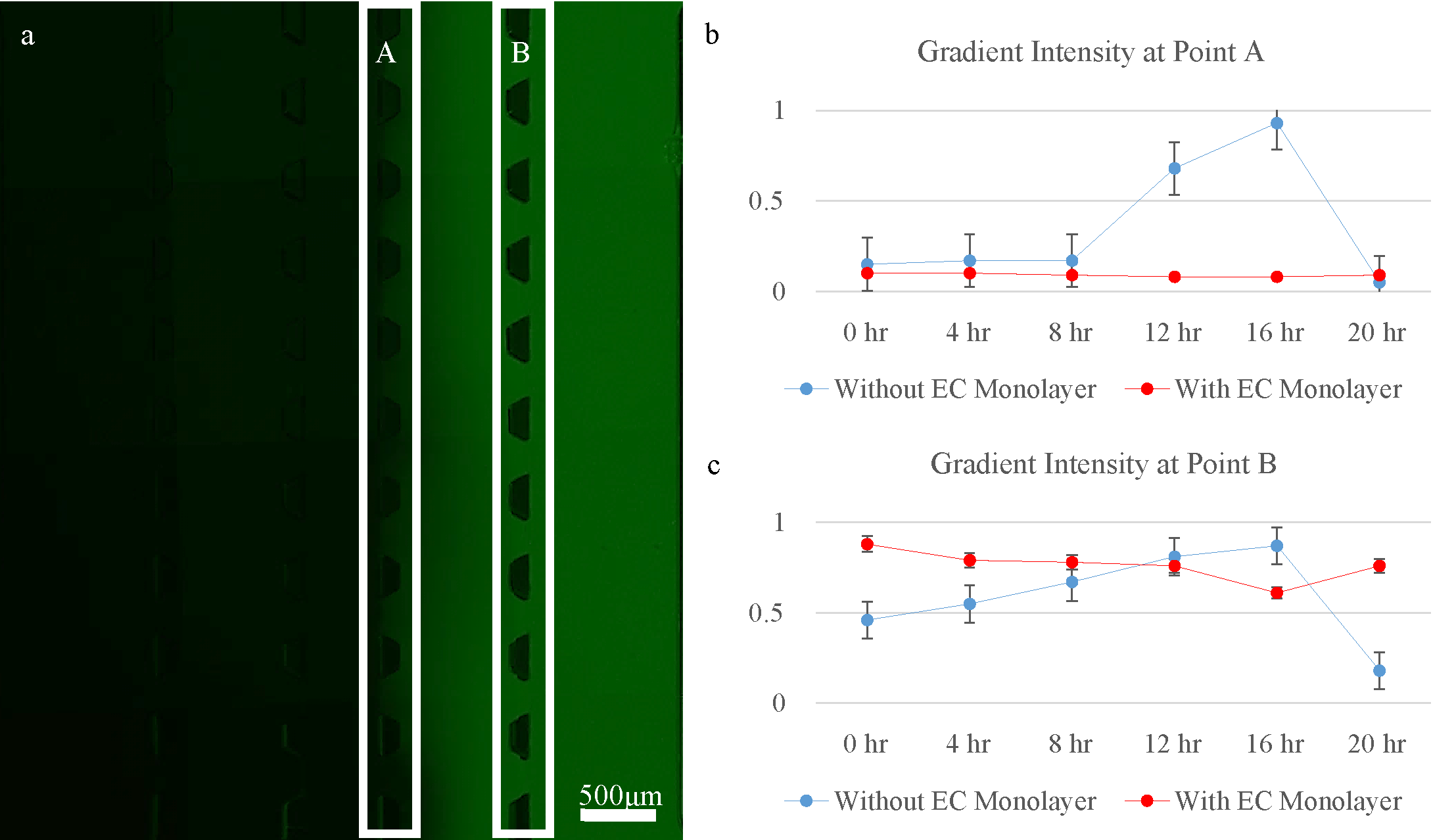

Supplement: S4 Fig — (a) The microfluidic device with a FITC-dextran gradient. White dashed box shows the region of interest for plot profiling of fluorescence intensity. Areas A and B are chosen to test the effects of endothelial barrier properties. (b) Gradient intensity at point A is shown on a graph. Notice that the fluorescent intensity is more stable when an endothelial monolayer is embedded. (c) Gradient intensity at Point B is shown on a graph, demonstrating a similar trend of gradient formation to Point A. (TIF) [file pone.0184595.s004.tif]

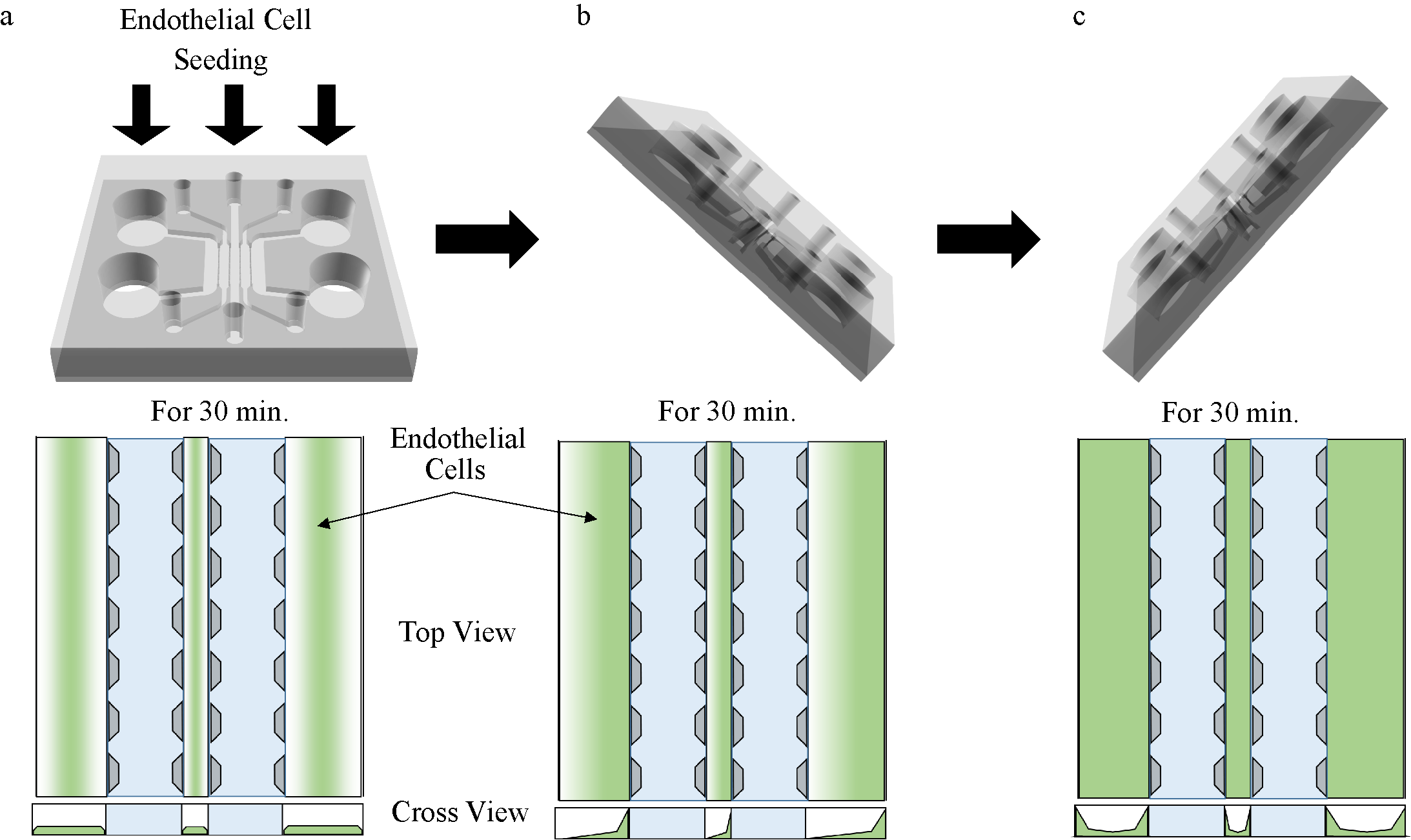

Supplement: S5 Fig — (a) ECs were introduced in the microfluidic device which was initially positioned upright. (b) The microfluidic device was skewed for endothelial monolayer’s confluence with collagen matrix. (c) The microfluidic device was skewed in the opposite direction for endothelial monolayer’s confluence with collagen matrix on both sides. The microfluidic device was kept in each position for 30 minutes each. (TIF) [file pone.0184595.s005.tif]
